# Supplementary material for: Lower limb muscle fatigue after uphill walking in children with unilateral spastic cerebral palsy
Source: PLoS One. 2022 Dec 6;17(12):e0278657. doi: 10.1371/journal.pone.0278657 (PMC9725134; doi:10.1371/journal.pone.0278657)
Supplement: S1 Text — (DOCX) [file pone.0278657.s001.docx]

# Appendix

## S1: Technical details Delsys Trigno sEMG sensors

| **Delsys Trigno Sensors** | |
| --- | --- |
| Dimension of a sensor | 27 x 37 x 15 mm |
| Mass of a sensor | 14.7 g |
| Sampling rate | 2000 samples/sec |
| Number of contacts (per sensor) | 4 |
| Contact dimensions | 5 x 1 mm |
| Inter electrode (contact) spacing | 10 mm |
| Contact material | 99.9% silver |
| Inter-sensor latency | <500 us (<1 sample period) |
| EMG Signal Bandwidth | 20-450 Hz |
| EMG Signal Sampling Rate | 2000 samples/sec |
| EMG baseline noise | <750 nV RMS |
| EMG Signal Resolution | 16 bit |
| **EMG analog output specifications** | |
| Number of outputs | 16 |
| Signal group delay | 48 ms |
| EMG Signal Range | ±5 V |
| Effective EMG Signal Gain | 909 V/V ±5% |
| Channel Offset | ±157 mV (max) |
| Baseline Noise | <0.5mV RMS |
| DAC Filter Badnwidth | DC-500 Hz, 160 dB/Dec |
| Passband Ripple | <2% with Sin(X)/X correction |
| Connector Type | SCSI-68, Type II |

## S2: SnPM figures

**SnPM t values (y-axis) during the gait cycle (% on x-axis) per joint**

| 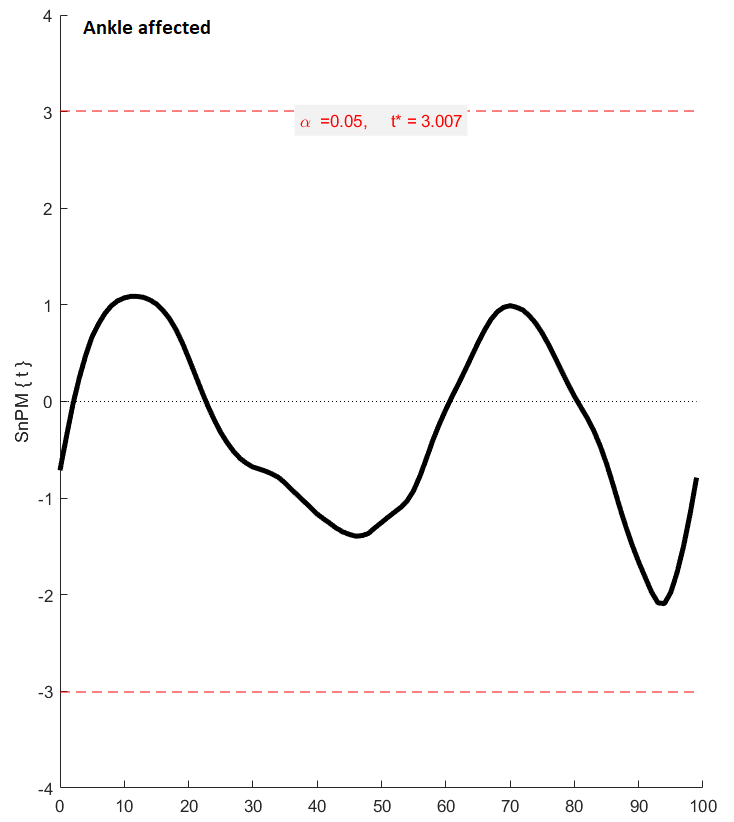 | 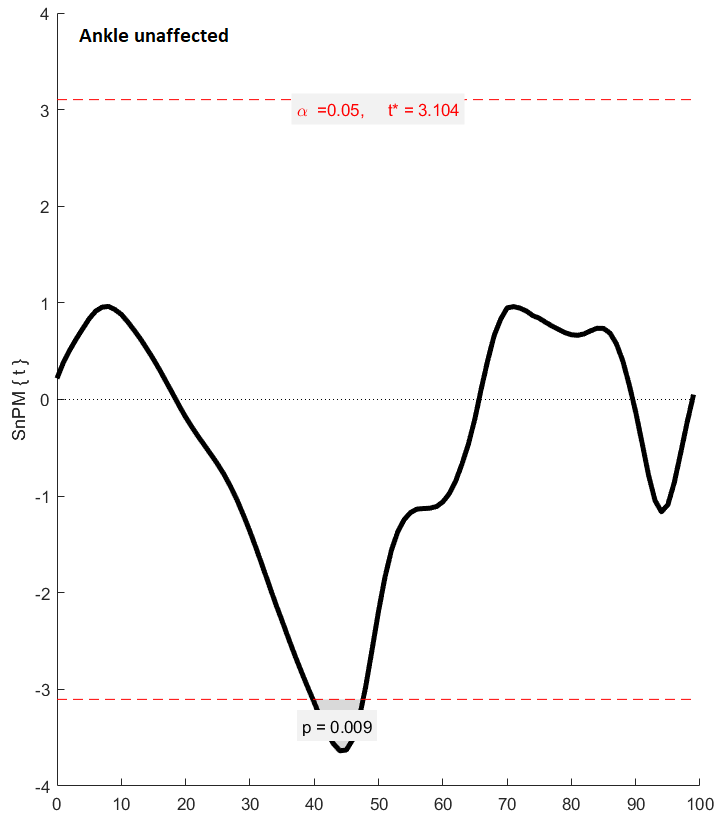 |
| --- | --- |
| 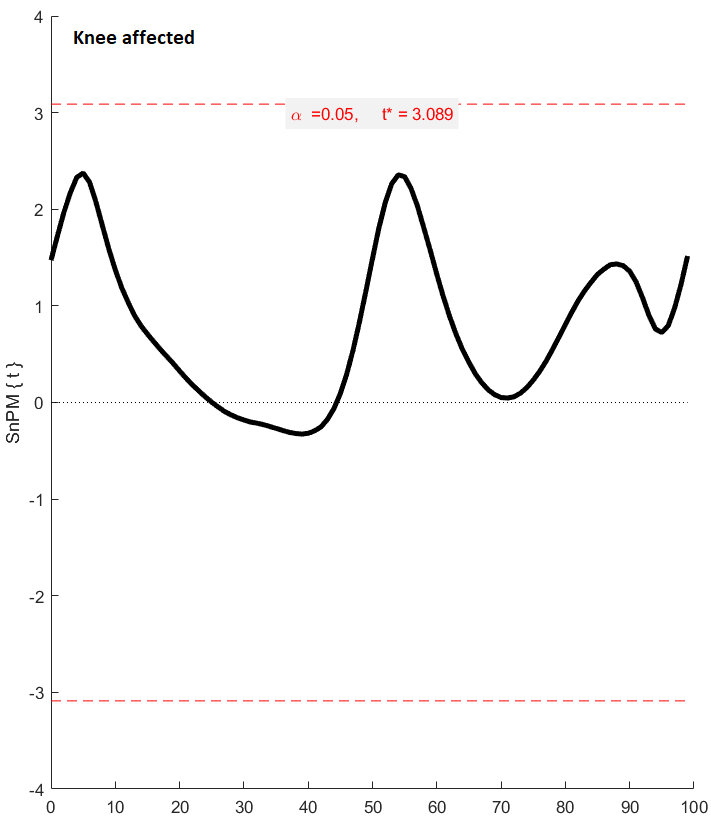 | 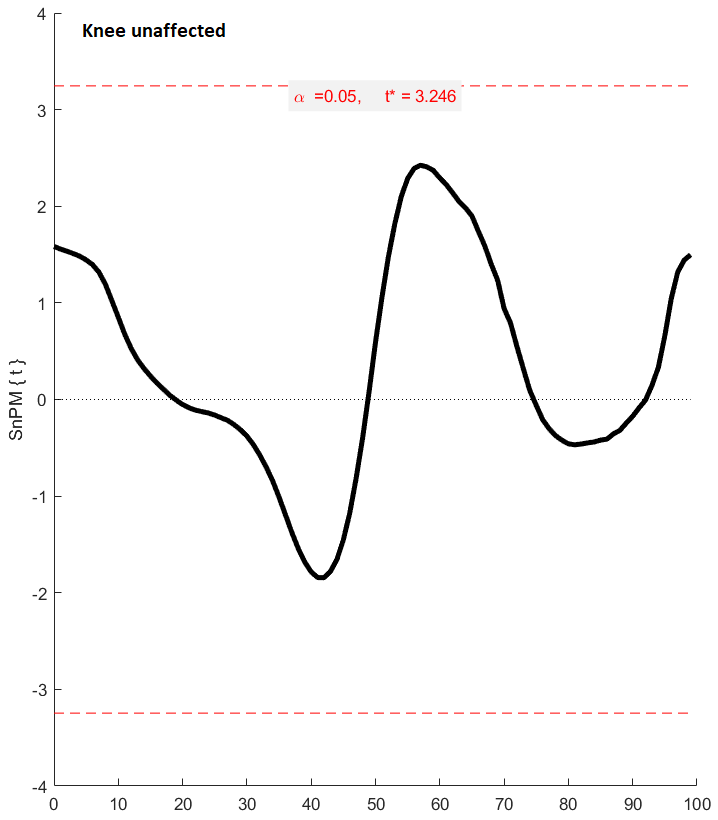 |
| 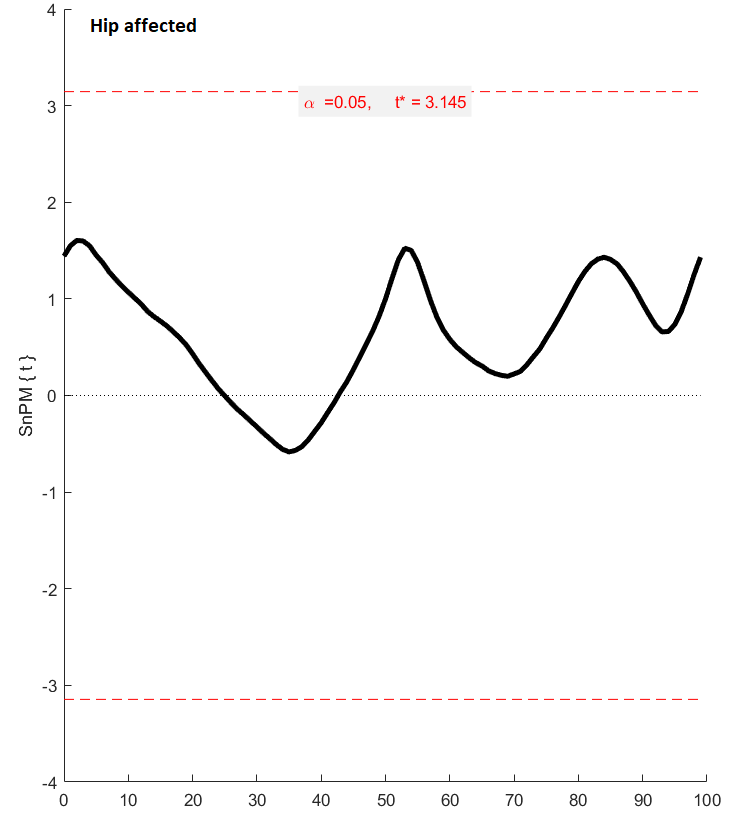 | 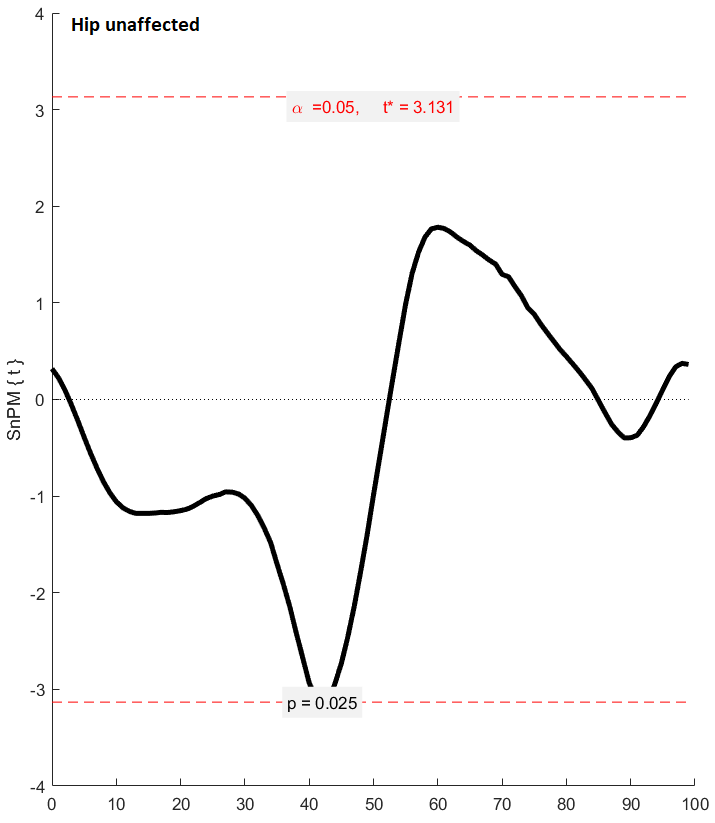 |

**Figure S2** SnPM t values (y-axis) during the gait cycle (% on x-axis) per joint. Ankle unaffected: statistically significant difference from 40-47.5% of the gait cycle: 1.2 to 1.4° more dorsiflexion in fatigued condition. Hip unaffected: statistically significant difference from 41.9 to 42.1% of the gait cycle: 1.0° less extension in fatigued condition. No statistically significant differences were found for the other joints.

## S3: Number of valid gait cycles per patient per condition

| **Patient number** | **F-Patient number**** | **Affected side** | **Condition** | **Number of valid gait cycles** |
| --- | --- | --- | --- | --- |
| 1 | 2 | Right | Comfortable | 127 |
| 1 | 2 | Right | Fatigued | 62 |
| 2 | 3 | Left | Comfortable | 127 |
| 2 | 3 | Left | Fatigued | 149 |
| 3* | 4* | Left | Comfortable | 129 |
| 3* | 4* | Left | Fatigued | 128 |
| 4 | 5 | Left | Comfortable | 127 |
| 4 | 5 | Left | Fatigued | 128 |
| 5 | 6 | Left | Comfortable | 129 |
| 5 | 6 | Left | Fatigued | 130 |
| 6 | 8 | Right | Comfortable | 128 |
| 6 | 8 | Right | Fatigued | 121 |
| 7* | 9* | Right | Comfortable | 127 |
| 7* | 9* | Right | Fatigued | 121 |
| 8* | 10* | Left | Comfortable | 136 |
| 8* | 10* | Left | Fatigued | 131 |
| 9 | 12 | Right | Comfortable | 138 |
| 9 | 12 | Right | Fatigued | 129 |
| 10 | 13 | Left | Comfortable | 133 |
| 10 | 13 | Left | Fatigued | 131 |
| 11 | 14 | Right | Comfortable | 146 |
| 11 | 14 | Right | Fatigued | 159 |
| 12 | 18 | Left | Comfortable | 130 |
| 12 | 18 | Left | Fatigued | 128 |
| 13* | 19* | Left | Comfortable | 128 |
| 13* | 19* | Left | Fatigued | 144 |
| 14 | 20 | Right | Comfortable | 128 |
| 14 | 20 | Right | Fatigued | 128 |
| 15 | 21 | Right | Comfortable | 133 |
| 15 | 21 | Right | Fatigued | 130 |
| 16 | 23 | Right | Comfortable | 138 |
| 16 | 23 | Right | Fatigued | 129 |
| 17* | 24* | Left | Comfortable | 129 |
| 17* | 24* | Left | Fatigued | 129 |
| 18 | 25 | Left | Comfortable | 136 |
| 18 | 25 | Left | Fatigued | 140 |

* = measurement 2 was used due to more missing data in measurement 1 (due to sensors that fell off or poor SNR). ** = the original patient number, also used in the individual kinematic Excel files (Supplementary data).
